# *Wolbachia* have made it twice: Hybrid introgression between two sister species of *Eurema* butterflies

**DOI:** 10.1002/ece3.6539

**Published:** 2020-07-08

**Authors:** Mai N. Miyata, Masashi Nomura, Daisuke Kageyama

**Affiliations:** ^1^ Graduate School of Horticulture Chiba University Matsudo Japan; ^2^ Institute of Agrobiological Sciences National Agriculture and Food Research Organization Tsukuba Japan

**Keywords:** cytoplasmic incompatibility, *Eurema hecabe*, *Eurema mandarina*, feminization, hybrid introgression, meiotic drive, mitochondrial DNA, *Wolbachia*

## Abstract

*Wolbachia*, cytoplasmically inherited endosymbionts of arthropods, are known to hijack their host reproduction in various ways to increase their own vertical transmission. This may lead to the selective sweep of associated mitochondria, which can have a large impact on the evolution of mitochondrial lineages. In Japan, two different *Wolbacahia* strains (*w*CI and *w*Fem) are found in two sister species of pierid butterflies, *Eurema mandarina* and *Eurema hecabe*. In both species, females infected with *w*CI (C females) produce offspring with a nearly 1:1 sex ratio, while females infected with both *w*CI and *w*Fem (CF females) produce all‐female offspring. Previous studies have suggested the historical occurrence of hybrid introgression in C individuals between the two species. Furthermore, hybrid introgression in CF individuals is suggested by the distinct mitochondrial lineages between C females and CF females of *E. mandarina*. In this study, we performed phylogenetic analyses based on nuclear DNA and mitochondrial DNA markers of *E. hecabe* with previously published data on *E. mandarina*. We found that the nuclear DNA of this species significantly diverged from that of *E. mandarina*. By contrast, mitochondrial DNA haplotypes comprised two clades, mostly reflecting *Wolbachia* infection status rather than the individual species. Collectively, our results support the previously suggested occurrence of two independent historical events wherein the cytoplasms of CF females and C females moved between *E. hecabe* and *E. mandarina* through hybrid introgression.

## Graphical abstract

Two sister species of butterflies, *Eurema mandarina* and *E. hecabe*, are distinct by nuclear gene sequences. The comparison of mitochondrial gene sequence variations revealed two independent occurrences of *Wolbachia*‐associated hybrid introgression.

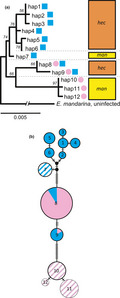

## 1 INTRODUCTION

The gram‐negative α‐proteobacteria, *Wolbachia*, are known as intracellular endosymbionts harbored by more than 40% of terrestrial arthropod species (Zug & Hammerstein, 2012). To enhance their transmission, *Wolbachia* selfishly manipulate their host's reproduction that can be broadly categorized as cytoplasmic incompatibility (CI) and female‐biased sex ratio distortion. CI is the most common phenotype occurring widely in terrestrial arthropods, wherein uninfected females are reproductively incompatible with infected males due to developmental arrest in their offspring at early embryogenesis, while infected females are compatible with both infected and uninfected males, allowing them to spread rapidly in host populations (Werren, Baldo, & Clark, 2008). Female‐biased sex ratio distortion can be achieved by inducing parthenogenesis in haplodiploid wasps, thrips, and mites, as well as male killing, feminization, or other mechanisms in a wide variety of diploid arthropods (Werren et al., 2008).

As the maternally transmitted *Wolbachia* spread, the hitchhiking effect allows the associated mitochondria to spread in the host population (Galtier, Nabholz, GlÉmin, & Hurst, 2009; Hurst & Jiggins, 2005). Previous studies suggested that mitochondrial DNA (mtDNA) diversity in *Wolbachia*‐infected individuals is typically lower than in uninfected individuals (Atyame, Delsuc, Pasteur, Weill, & Duron, 2011; Avtzis, Doudoumis, & Bourtzis, 2014; Jiang et al., 2014; Raychoudhury et al., 2010; Schuler et al., 2016; Shoemaker, Dyer, Ahrens, McAbee, & Jaenike, 2004; Turelli, Hoffmann, & McKechnie, 1992; Xiao et al., 2012). Furthermore, *Wolbachia* may facilitate mtDNA introgression in closely related host species following hybridization (Charlat et al., 2009; Dyer, Burke, & Jaenike, 2011; Jäckel, Mora, & Dobler, 2013; Jiggins, 2003; Narita, Nomura, Kato, & Fukatsu, 2006; Rousset & Solignac, 1995).

The common yellow butterfly, *Eurema mandarina* (Lepidoptera: Pieridae) [former name: *Eurema hecabe*, yellow type], is widely distributed over the Japanese archipelago. *Eurema hecabe* [former name: *Eurema hecabe*, brown type], a sister species of *E. mandarina* is found to be widely distributed in Asia, Africa, Australia, Okinawa‐jima Island and other islands south of it (Yata, 1989, 1995). Two different strains of *Wolbachia*, *w*CI (ST41) and *w*Fem (ST40) (Baldo et al., 2006), have been characterized from *E. mandarina* (Hiroki, Tagami, Miura, & Kato, 2004) and *E. hecabe* (Narita et al., 2011). In both butterfly species, individuals infected with *w*CI (C individuals) exhibit CI, whereas individuals infected with both *w*CI and *w*Fem (CF individuals) produce only daughters (Hiroki, Kato, Kamito, & Miura, 2002; Hiroki et al., 2004; Narita et al., 2011). A recent study revealed that, in *E. mandarina*, the sex chromosome composition of C males, C females, and CF females are ZZ, ZW, and Z0, respectively (Kageyama et al., 2017; Kern, Cook, Kageyama, & Riegler, 2015). *Wolbachia* disrupt the maternal inheritance of the Z chromosome in Z0 individuals and *Wolbachia* is likely to play a female‐determining role, like the W chromosome in Z0 individuals that would be otherwise determined as male in the absence of *Wolbachia* (Kageyama et al., 2017). However, it has not been clarified whether the meiotic drive is the mechanism behind the all‐female trait in *E. hecabe*.

Although Z‐linked nuclear DNA (ncDNA) clearly discriminated *E. mandarina* and *E. hecabe*, mitochondrial lineages are largely clustered into two groups, according to the *Wolbachia* infection status rather than the species—one consisting of uninfected (*Wolbachia*‐free) *E. mandarina* and the other of *Wolbachia*‐infected *E. mandarina* and *E. hecabe* (Miyata, Konagaya, Yukuhiro, Nomura, & Kageyama, 2017; Narita et al., 2006; Narita, Nomura, Kato, Yata, & Kageyama, 2007). These results strongly support the historical occurrence of cytoplasmic introgression from *E. hecabe* to *E. mandarina* (Narita et al., 2006). Closer inspection using appropriate mtDNA markers revealed that the *Wolbachia*‐infected mitochondrial lineages of *E. mandarina* were slightly but clearly differentiated into two clusters depending on the presence or absence of *w*Fem (Miyata et al., 2017). This result raises the possibility that cytoplasmic introgression from *E. hecabe* into *E. mandarina* occurred at least twice over their evolutionary history (Miyata et al., 2017).

To verify this possibility, we sequenced ncDNA and mtDNA fragments of *E. hecabe* collected on Ishigaki‐jima Island and subjected them to phylogenetic analyses and haplotype network analyses, together with the previously published sequence data of *E. mandarina*(Miyata et al., 2017). We specifically aimed to clarify the following evolutionary questions: (a) Did *Wolbachia* infection affect mtDNA diversity of *E. hecabe*? (b) How did the two *Wolbachia* strains (*w*CI and *w*Fem) move between *E. mandarina* and *E. hecabe*?

## 2 METHODS

### 2.1 DNA extraction

**Figure 1:**
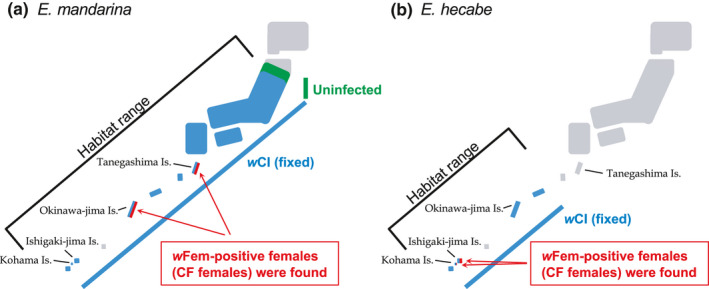
Habitat range and *Wolbachia* infection status of *E. mandarina* and *E. hecabe* in Japan

In total, 61 adult females of *E. hecabe* were collected on Ishigaki‐jima Island (Okinawa prefecture, Japan; N24°20.04′, E124°09.22′) (Figure 1) between 2015 and 2017 and stored at −30°C until further analysis. The thoracic muscles of *E. hecabe* were subjected to DNA extraction using a DNeasy Blood & Tissue Kit (Qiagen).

### 2.2 Diagnostic PCR and sequencing of *Wolbachia wsp* gene

The extracted DNA was subjected to diagnostic PCR targeting the *wsp* gene to detect either *w*CI or *w*Fem strains of *Wolbachia* (Kageyama, Narita, & Noda, 2008; Kageyama et al., 2017; Miyata et al., 2017; Narita, Kageyama, Nomura, & Fukatsu, 2007). Specifically, *w*CI was detected using the *Wolbachia*‐specific forward primer wsp81F (5′–TGGTCCAATAAGTGATGAAGAAAC–3′) (Braig, Zhou, Dobson, & O’Neill, 1998; Zhou, Rousset, & O’Neil, 1998) and the *w*CI‐specific reverse primer WHecFem1 (5′–ACTAACGTTTTTGTTTAG–3′) (Hiroki et al., 2004) which amplify the 232‐bp DNA fragment, while *w*Fem was detected using the *w*Fem‐specific forward primer WHecFem2 (5′–TTACTCACAATTGGCTAAAGAT–3′) (Hiroki et al., 2004) and the *Wolbachia*‐specific reverse primer wsp691R (5′–AAAAATTAAACGCTACTCCA–3′) (Braig et al., 1998; Zhou et al., 1998) which amplify the 398‐bp DNA fragment. For both reactions, the following temperature profile was adopted: 35 cycles of denaturation at 95°C for 1 min, annealing at 58°C for 1.5 min, extension at 72°C for 1.5 min, and a final extension at 72°C for 7 min.

For the characterization of *Wolbachia* strains, the PCR products of the *wsp* gene were used for DNA sequencing. The PCR products purified by ExoSAP‐IT (Usb) were directly sequenced using a BigDye Terminator version 3.1 Cycle Sequencing Kit (Thermo Fisher Scientific K.K.) and analyzed with an ABI PRISM 3130xl Genetic Analyzer (Applied Biosystems Inc.).

### 2.3 PCR and sequencing of host ncDNA and mtDNA genes

To amplify the highly variable intron of the Z‐linked triosephosphate isomerase (*Tpi*) gene, we used the following primers: 5′–GGTCACTCTGAAAGGAGAACCACTTT–3′ and 5′‐CACAACATTTGCCCAGTTGTTGCAA–3′, located in coding regions (Jiggins et al., 2001). For mtDNA genes, the cytochrome *c* oxidase subunit I (*COI*) gene was amplified using C1–J–1718 (5′–GGGGGGTTTGGAAATTGATTAGTGCC–3′) and TL2–N–3014 (5′–TCCATTGCACTAATCTGCCATATTA–3′) (Simon et al., 1994). Cytochrome *c* oxidase subunit III (*COIII*) and the adjacent *t‐RNA* gene were amplified using COX3–F2 (5′–TCAGCTGTTGCTATAATTCAA–3′) and COX3–R2 (5′–TATGATTGGAAGTCAAATATA–3′) (designed by Naoto Haruyama). The PCR conditions were 94°C for 5 min, followed by 35 cycles of denaturation at 94°C for 30 s, annealing at X°C for 30 s and extension at 72°C for 30 s, and final extension at 72°C for 7 min, where X was 42.6 for *COI*, 44.4 for *COIII* and adjacent *t‐RNA* and 48.7 for *Tpi*. The PCR products purified by ExoSAP‐IT were subjected to a sequencing reaction using a BigDye Terminator v3.1 Cycle Sequencing Kit. The results were then analyzed by an ABI PRISM 3130xl Genetic Analyzer. For those samples that failed to sequence *COI*, PCR products were gel‐excised and purified using a QIAquick Gel Extraction Kit (Qiagen) before the sequencing reaction.

### 2.4 Molecular phylogenetic analyses

Phylogenetic trees were constructed using the maximum‐likelihood method on MEGA software version 7.0.26 (Kumar, Stecher, & Tamura, 2016). Aligned nucleotide sites containing gaps were removed. The most suitable nucleotide substitution model was selected using the Find Best DNA Model implemented in MEGA. For this analysis, the ML tree of the *Tpi* sequence was inferred using the selected Tamura 3‐parameter model with a gamma distribution, while the ML tree of concatenated sequences of *COI* and *COIII* were inferred using the selected Hasegawa–Kishino–Yano model with gamma distribution. *Eurema blanda* was used as an out‐group. An mtDNA haplotype network was generated with TCS software version 1.21 (Clement, Posada, & Crandall, 2000). The TCS program calculates the minimal number of mutation steps by which the sequences can be joined with > 95% confidence. We added the previously published sequence data for *E. mandarina* (based on 30 adult females) (Miyata et al., 2017) to both analyses. Additionally, we performed Tajima's *D* test and Fu and Li's *F* and *D* test using DnaSP version 5 (Librado & Rozas, 2009). The nucleotide sequences of *Tpi, COI*, and *COIII* and adjacent *t‐RNA* genes of *E. hecabe, E. mandarina,* and *E. blanda* were deposited in the DDBJ/EMBL/GenBank databases under accession numbers LC468246‐LC468418, LC511749, and LC511750.

## 3 RESULTS

### 3.1 Nuclear *Tpi*

**Figure 2:**
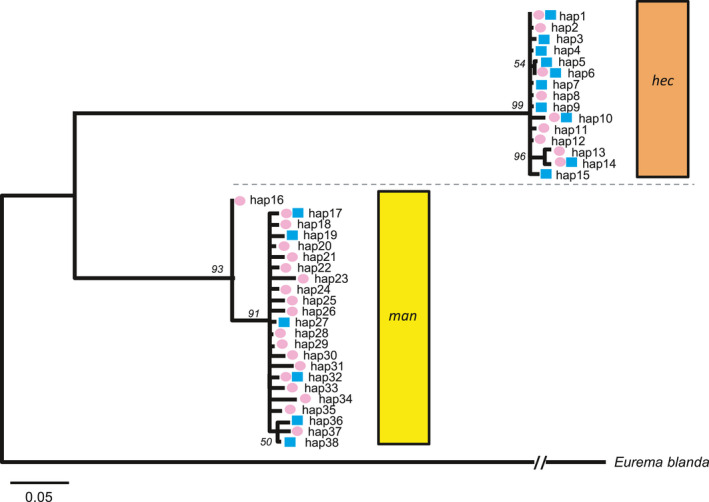
Maximum likelihood tree of *E. mandarin* *a* and *E. hecab* *e* based on nuclear *Tpi* sequences. Pink ovals, CF females; blue rectangles, C females

Among the 61 *E. hecabe* individuals (30 CF and 31 C), partial sequences of the intronic region (413 bp) of the nuclear *Tpi* gene were polymorphic in 27 nucleotide sites, constituting 15 haplotypes. Based on these sequences and the *E. mandarina Tpi* dataset published previously (Miyata et al., 2017), the ML tree of the *Tpi* sequence (ln(*L*) = −1,906.27 according to the Tamura 3‐parameter model with gamma distribution) was constructed. The tree was topologically split into two clades; one composed of *E. hecabe*, the other of *E. mandarina* (Figure 2). In each species, there were no fixed substitutions in the *Tpi* sequences distinguishing between C and CF females.

### 3.2 Mitochondrial *COI* and *COIII*

**Figure 3:**
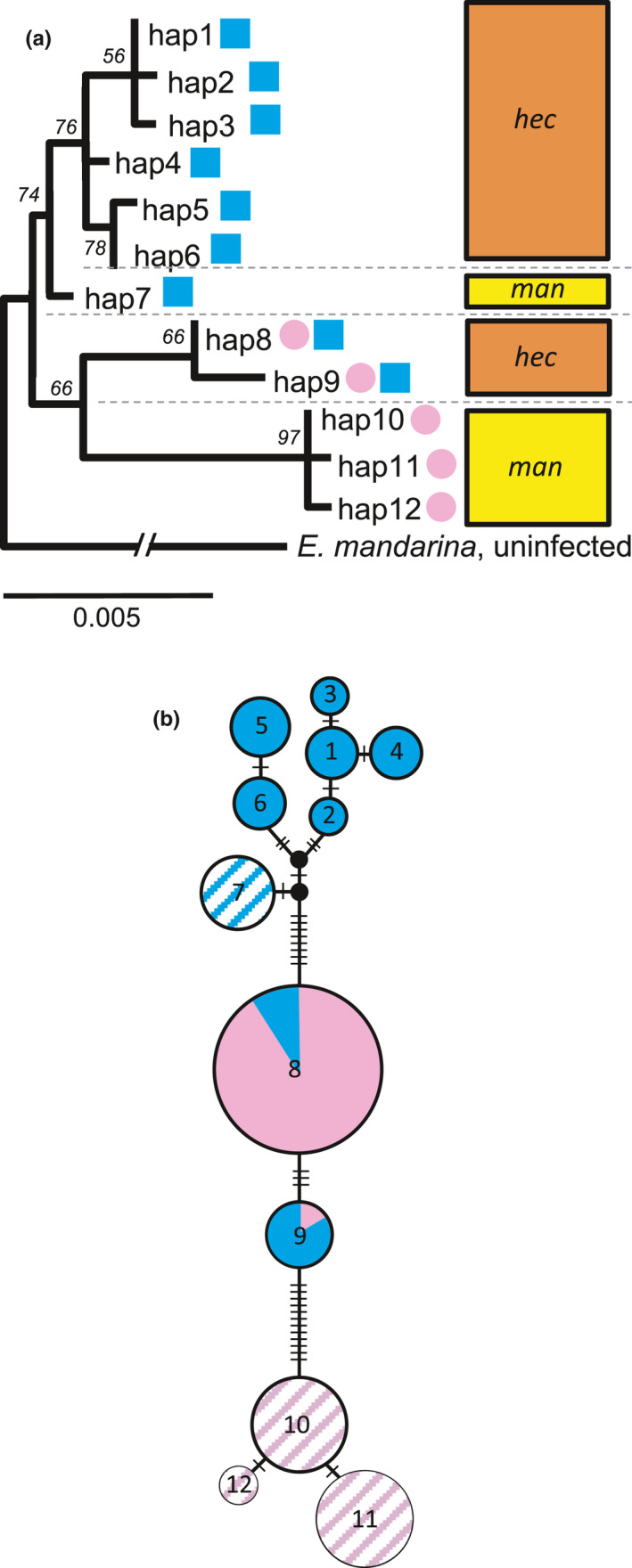
Molecular phylogenetic analyses based on mitochondrial *CO* *I* and *COIII* sequences of *E. mandarina* and *E. hecabe*. (a) Maximum likelihood tree. (b) TCS parsimony network. Area of each circle and square is proportional to the number of individuals. Filled circles, *E. hecabe*; Striped circles, *E. mandarina*;Pink, CF females; blue, C females

Among the 73 individuals of *E. hecabe* (26 CF and 22 C) and *E. mandarina* (19 CF and 6 C), concatenated sequences of *COI* and *COIII*, including adjacent *t‐RNA* (2,023 bp in total), were polymorphic in 27 nucleotide sites, constituting 12 haplotypes. *E. mandarina* fell into four haplotypes (haplotypes 3, 4, and 5 consisted of CF individuals and haplotype 6 consisted of C individuals; Figure 3a,b). *E. hecabe* fell into 8 haplotypes (haplotypes 1 and 2 consisted of both C and CF individuals, while haplotypes 7, 8, 9, 10, 11, and 12 consisted of only C individuals; Figure 3a,b). On the ML tree of concatenated sequences (ln(*L*) = −3,097.08 according to the Hasegawa–Kishino–Yano model with gamma distribution), the monophyly of C haplotypes (haplotypes 1–7) was supported by a bootstrap value of 74%, while that of CF‐containing haplotypes (haplotypes 8–12) was supported by a bootstrap value of 66% (Figure 3a). Among the C clade (haplotypes 1–7), *E. hecabe* formed a monophyletic group (haplotypes 1–6), which was distinct from *E. mandarina* (haplotype 7). On the other hand, the CF‐containing clade (haplotype 8–12) was divided into two clades: one consisting of *E. hecabe* (haplotypes 8 and 9) and the other of *E. mandarina* (haplotypes 10–12). Although estimates of Tajima's *D* did not imply significant deviation from neutral expectation in any of the categories, estimates of Fu and Li's *F* and *D* statistics implied a recent selective sweep in *E. hecabe* CF individuals (Fu and Li's *F* = −2.70880, *p* < .05; Fu and Li's *D* = −2.58495, *p* < .05) (Table 1).

**Table 1:** Neutrality test for *E. mandarina* and *E. hecabe* from mtDNA sequence

Species	Infection status	*N*	Tajima's *D*	Fu and Li's *F*	Fu and Li's *D*
*E. mandarina*	C	6	cannot be calculated	cannot be calculated	cannot be calculated
	CF	19	0.2479	−0.4051	−0.5736
*E. hecabe*	C	22	1.70080	1.62637*	1.26534
	CF	26	−1.73363	−2.70880*	−2.58495*
Total		73	2.04512	1.83119*	1.22893

* *p* < .05.

## 4 DISCUSSION

Our phylogenetic analyses using ncDNA and mtDNA sequences of the two *Eurema* butterflies containing both C and CF females suggested the occurrence of two independent hybrid introgression events—one involving the maternal lineages infected with *w*CI and the other involving maternal lineages infected with both *w*CI and *w*Fem.

Two independent occurrence of hybrid introgression comes from the supposition that mitochondria of CF and C individuals of the two species are each likely to be descendants of common ancestors. Although each formed a clade, bootstrap values of the C‐ and CF‐containing clades were not very high (C clade: 74%; CF clade: 66%). Higher bootstrap support may be obtained by using more informative mtDNA sites with a larger sample size. Alternatively, a more complex evolutionary history might be hidden in these butterflies such as involvement with other species not endemic to Japan.

The occurrence of *Wolbachia*‐mediated hybrid introgression has been described in various insect species (Dyer et al., 2011; Gaunet et al., 2019; Jäckel et al., 2013; Jiggins, 2003; Lachaise et al., 2000; Raychoudhury, Baldo, Oliveira, & Werren, 2009; Sahoo et al., 2018; Turelli et al., 1992, 2018), but our study is unique in the sense that *Wolbachia* have different impacts on their hosts (e.g., CI and meiotic drive), which independently induced hybrid introgression between the same butterfly species *E. mandarina* and *E. hecabe*.

Hiroki and Kato (2006) demonstrated that F1 females generated by the mating between an *E. mandarina* female and an *E. hecabe* male possessed immature ovaries; in the reciprocal cross (i.e., the mating between *E. hecabe* female and *E. mandarina* male), however, F1 females possessed fully developed ovaries, even in young adults (Hiroki & Kato, 2006). Taking these observations into consideration, it is reasonable to assume that the cytoplasm of CF individuals, as well as C females, moved from *E. hecabe* into *E. mandarina*. Considering that the mtDNA of CF individuals is more deeply branched between the two species compared to that of C individuals (Figure 3a), hybrid introgression may have occurred earlier in CF individuals than in C individuals.

We should mention here that our results do not concur with the conclusions of Narita, Nomura, Kato, et al. (2007), who found a lack of association between *Wolbachia* infection status (C vs. CF) and mtDNA haplotypes in *E. hecabe* collected in Southeast Asia. We aligned our sequences together with the published sequences of Narita, Nomura, Kato, et al. (2007) and performed a phylogenetic analysis for this dataset (Additional file 1). Although the resolution of the phylogenetic tree was not high, partly due to the lack of the highly variable region in 3ʹ‐end of *COIII*, *Eurema* butterflies in Southeast Asia seem to have a much higher mtDNA diversity than their Japanese counterparts. However, the phylogenetic tree did not reflect *Wolbachia* infection status. According to this tree, C and CF lineages of *E. hecabe* in Ishigaki‐jima Island may be the descendants of randomly sampled founders due to genetic drift. Caution should also be paid for the possibility that some of the samples in Narita, Nomura, Kato, et al. (2007) were not of *E. hecabe* but a cryptic species; although they were identified by morphological observation, no ncDNA was sequenced for those samples. Nevertheless, our study has provided a likely scenario that C and CF lineages of *E. mandarina* share common ancestors with C and CF lineages of *E. hecabe*, respectively, and that each of them independently experienced hybrid introgression.

The fact that mtDNA haplotypes of in *E. hecabe* CF females were also found in a small number of C females may suggest that the vertical transmission of *w*Fem is imperfect in this species. In *E. mandarina*, (Narita, Nomura, and Kageyama (2007) found that the vertical transmission rates of *w*CI and *w*Fem were 100% and 80%, respectively. Although the offspring sex ratio of CF females that selectively lost *w*Fem is still unknown, the elimination of *Wolbachia* from *E. mandarina* CF females led to the production of male offspring only (Hiroki et al., 2002; Kageyama et al., 2017). As cytoplasm is only transmitted maternally, this result may explain the absence of haplotypes shared by CF and C females in *E. mandarina*. In this sense, the shared mtDNA haplotypes between C females and CF females of *E. hecabe* might suggest a different mechanism of all‐female production between *E. mandarina* and *E. hecabe*. It should be noted that the effect of *Wolbachia* elimination on sex ratio still remains to be examined in *E. hecabe*.

The estimates of Fu and Li's *F* and *D* statistics (Table 1) suggested that the *E. hecabe* CF females experienced recent population expansion, purifying selection, or genetic hitchhiking. It is possible that CF individuals recently increase their frequency in the population of *E. hecabe*. It will be of interest to examine, in our future studies, population dynamics regarding sex ratio and *Wolbachia* infection of *E. hecabe* in Ishigaki‐jima Island, which can be compared with the data of *E. mandarina* on Tanegashima Island (Kageyama et al., 2020) in terms of population ecology and evolutionary biology.

## CONFLICT OF INTEREST

The authors have no competing interests.

## AUTHOR CONTRIBUTION

**Mai N. Miyata:** Conceptualization (equal); Data curation (lead); Formal analysis (lead); Funding acquisition (lead); Investigation (lead); Methodology (lead); Visualization (equal); Writing‐original draft (lead); Writing‐review & editing (equal). **Masashi Nomura:** Conceptualization (equal); Investigation (supporting); Methodology (supporting); Project administration (lead); Writing‐review & editing (equal). **Daisuke Kageyama:** Conceptualization (equal); Data curation (supporting); Formal analysis (supporting); Investigation (supporting); Methodology (supporting); Project administration (supporting); Writing‐review & editing (lead).

### OPEN RESEARCH BADGES

This article has earned an Open Data Badge for making publicly available the digitally‐shareable data necessary to reproduce the reported results. The data is available at https://osf.io/537gq/?view_only=6924c0a843af4f8aba167626a7111df7.

## Supporting information

Additional file 1

## Data Availability

Sample information are available on OSF (https://osf.io/537gq/?view_only=6924c0a843af4f8aba167626a7111df7).
