# Supplementary material for: Wolbachia have made it twice: Hybrid introgression between two sister species of Eurema butterflies
Source: Ecol Evol. 2020 Jul 8;10(15):8323–30. doi: 10.1002/ece3.6539 (PMC7417220; doi:10.1002/ece3.6539)
Supplement: Supplementary file 1 — Additional file 1 [file ECE3-10-8323-s001.pdf]

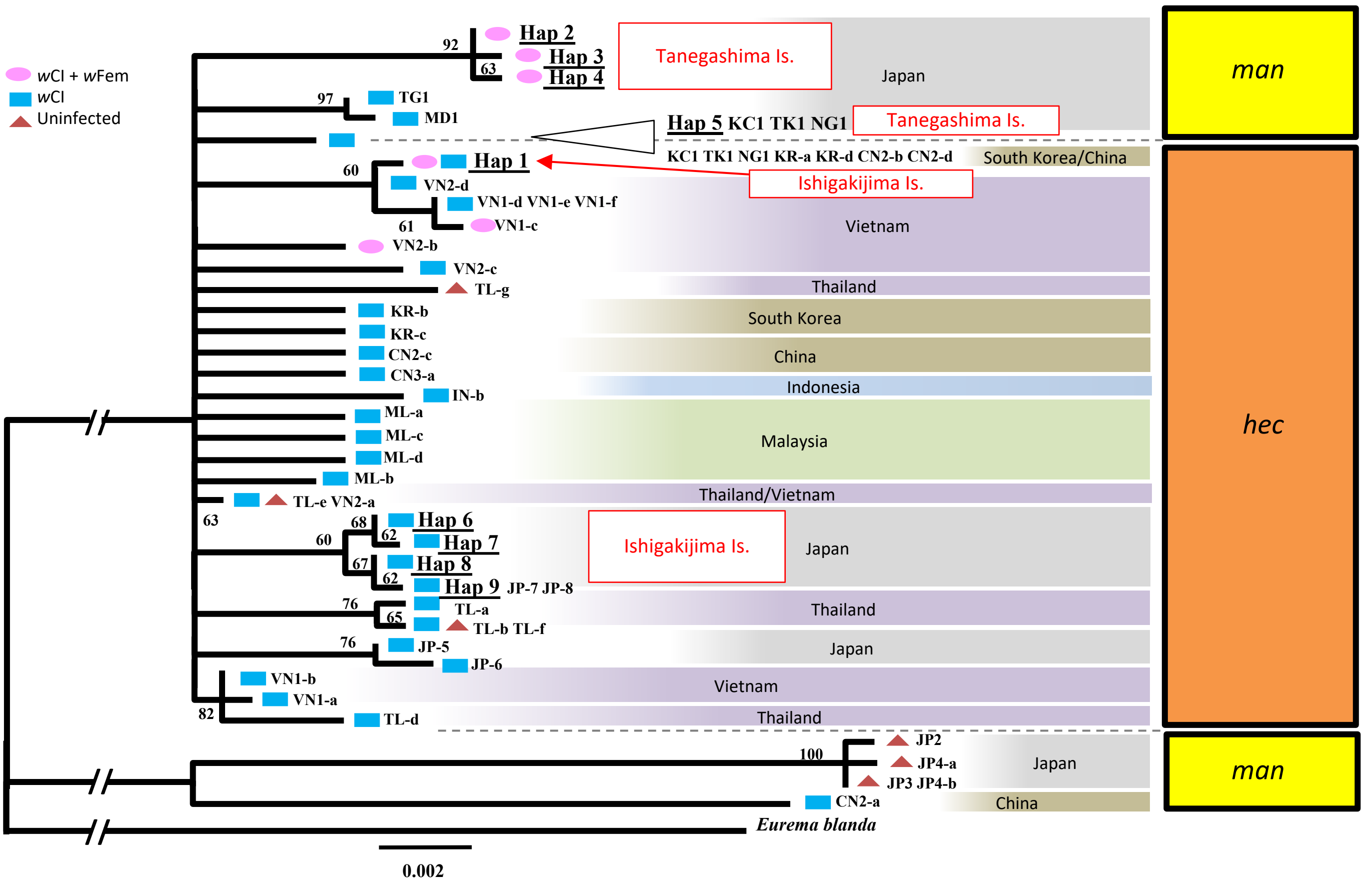

Addirional file1.

Maximum likelihood tree of *E. mandarina* and *E. hecabe* based on COI and COIII sequences obtained in this study together with the published sequences of Narita et al. (2007c).
